# Editorial: Characterisation, functions and roles of antigen-specific regulatory T cells in health and disease

**DOI:** 10.3389/fimmu.2022.1022813

**Published:** 2022-09-27

**Authors:** Giang T. Tran, Nirupama D. Verma, Lesley M. Smyth, Bruce M. Hall

**Affiliations:** ^1^ Immune Tolerance Laboratory, Ingham Institute for Applied Medical Research, University of New South Wales (UNSW), Sydney, NSW, Australia; ^2^ South Western Sydney Clinical School, University of New South Wales (UNSW), Sydney, NSW, Australia; ^3^ Department of Bioscience, School of Health, Sport and Bioscience, University of East London, London, United Kingdom

**Keywords:** Treg - regulatory T cell, antigen-specificity, immunotherapy, immune tolerance, Foxp 3

## Introduction

The control of immune responses against self and how to induce immune tolerance has intrigued immunologists for nearly 75 years. The clonal deletion theory of Burnet (1) received unquestioning support and is still a partial explanation for lack of reactivity to self.

A decade after the discovery that lymphocytes produced by the thymus are key to immune responses (2), Gershon et al. described thymus derived suppressor cells (3) and these cells were implicated in prevention of autoimmunity and transplant tolerance (4). In addition, neonatal and adult thymectomy promote the development of autoimmunity (4) which could be suppressed by transfer of normal lymphocytes (5). Studies on suppressor cells identified they were a subset of CD8^+^T cells that expressed I-J, a molecule associated with “Ia” in MHC (5). Cloning of mouse MHC found no gene for I-J in MHC of mice (6) and suppressor cells were declared non existent (7). The word “suppressor cells” was eliminated from the immunological dictionary.

From near extinction, suppressor cells that expressed CD4 not CD8 were discovered in the mid 1980 by Hall et al. and their activity was antigen-specific (8). These CD4^+^ regulatory cells had markers of activated/memory T cells, including the IL-2 receptor CD25, expression of lower molecular weight forms of CD45, and MHC class II (9). Furthermore, these cells are highly dependent on specific-antigen and cytokines to survive and expand (10) but IL-2 alone did not promote their survival. It was for this reason, Hall et al. looked for CD25 expression on antigen-specific suppressor cells. These antigen-specific T suppressor cells had other characteristics of an activated memory cells (11) in that they did not recirculated from blood to lymph (12). The inability of IL-2 alone to sustain activated antigen-specific CD4^+^CD25^+^T cells, led us to investigate the role of other cytokines in activation and maintenance of CD4^+^CD25^+^Treg (13, 14). We found that naïve Treg proliferated when cultured with either IL-2 or IL-4 in the presence of antigen acquired increased potency to suppress *in vitro* and *in vivo* (13).

Whilst these findings from the 1980s lay unrecognized, two studies reinvigorated research on regulatory cells. First the demonstration of infectious tolerance mediated by CD4^+^ T cells (15). Second, Sakaguchi’s application of Hall’s findings on activated CD4^+^CD25^+^ T cells to show they also prevent autoimmunity (16). The title of their paper starts as “Immunologic self-tolerance maintained by activated T cells expressing IL-2 receptor alpha-chains (CD25).” indicating they accepted our premise that CD4^+^CD25^+^ T cells were activated CD4^+^T cells. It is now clear the cells they described were resting, thymus derived cells whose TCR recognize autoantigens. These cells express FoxP3, the transcription factor that maintains regulatory function of Tregs (17). How naïve resting thymic derived Treg with no antigen specificity are activated to antigen-specific Tregs remains an unfolding puzzle. The potential of antigen specific Tregs as therapy in autoimmune disease and transplantation was rapidly appreciated and remains a subject of active investigation.

Articles in this Research Topic illustrate the wide range of studies being undertaken to improve our understanding of how antigen-specific Treg are activated, can be monitored, expanded and their potential application for therapies.

The role of cytokines in Treg activation is discussed in several papers. Bhaskaran et al. investigated the role of IL-1β -MyD88-mTor in activation of Th17-like Treg. MyD88 deletion in FoxP3^+^ T cells alters its function and results in increased mucosal infection and inflammation which coincided with the reduction of IL-17A expressing FoxP3^+^ (Treg17) and increased Treg IFN-γ. Hall et al describe how IL-5 therapy promotes the generation of a unique population of antigen-specific Tregs that is highly potent in prevention of allograft rejection. These cells are called Ts2 and have upregulation of IL-5Rα.

Iwaszkiewicz-Grzes et al described epigenetic changes with antigenic stimulation of Treg. They show Treg activation by specific antigen alters their functional capacity.

Shimojima et al. studied Treg stability in antibody associated vasculitis, showing inhibition of activated Tregs by oxidative stress, reinforcing the negative effect inflammation has on Treg function. The mediator was reactive oxygen species (ROS) acting *via* phosphorylated mammalian target of rampamycin (mTOR).

Shevyrev et al. reviewed the interaction of recognition and presentation in activation of T cells, including conversion of effector cells to regulatory cells.

The complexities of growing antigen-specific Treg are highlighted in three articles. Lee et al. described how they expand high potency human alloreactive Treg *ex vivo*. They compared expansion efficiency and characteristics of *ex vivo* expanded human Treg generated by stimulation with either, allogeneic stimulated B cells (sBcs) or matured monocyte derived dendritic cells (sDCs). Both protocols induced a similar Treg phenotype, but sDCs expanded twice the number of Treg. These findings establishe that sDCs stimulation is a viable option for alloreactive Treg expansion.

Cortes-Hernandez et al. described how antigen-specific Treg can be expanded from patients with renal failure. This study showed purification of alloantigen specific Treg from chronic kidney disease patients and their successful long-term expansion that maintained their suppressive phenotype and function.

Therapeutic potential of antigen-specific Treg is demonstrated in several papers. To circumvent the complexities of growing natural Treg, Muller et al. detailed methods to genetically engineer anti-HLA-A2 regulatory T cells as potential inducers of transplant tolerance. HLA-A2 mono-specific CAR Treg maintained Treg phenotype and function *in vitro* and *in vivo* as they selectively homed to HLA-A2 expressing islets grafts.

Selck and Dominguez-Villar reviewed approaches to generate antigen-specific Tregs by genetic engineering of antigen-specific T effector cells or polyclonal Tregs, and activation of Tregs *in vivo*.

The roles of activated Tregs in diseases such as pulmonary hypertension(Tian et al.) and therapy for food allergy (Liu et al.), milk intolerance (Zhang et al.), are reviewed with discussion of many important aspects of using antigen-specific therapies in autoimmune disease including using combined strategies and tissue specific targeting (Serr et al.). Hu et al. reviewed the role of antigen-specific Tregs in renal transplantation (Selck and Dominguez-Villar) .

Hu et al described cells that do not share standard markers of Treg but exhibit regulatory functions. They studied a possible mechanism of induction of previously described TCRαβ^+^double negative T cells (DNT) from CD4^+^ T cells by stimulation with immature DCs.

Liu et al studying chronic hepatitis B patients showed expression of NKG2D on iDNT cells enhanced their regulatory function of limiting proliferation and survival of B cells. IL-35 dependent T cells regulation by regulatory IL-35^+^B cells within classical CD19^+^CD24^hi^CD38^+^Breg is described in chronic hepatitis B, and was dependent on cell-to-cell contact controlling IFN-γ producing CD4^+^ and CD8^+^T cells.

Lyu et al. used single cell RNA sequencing and TCR sequencing to assess the functions of CD4^+^ T cell subsets, including Treg and their interactions, during CMV infection. Treg phenotype during CMV infection showed markers that are proinflammatory, inhibitory, chemokine receptors and cytotoxic related markers in addition to characteristic markers of Treg. This suggests clustering of these cells in a self-sustaining positive feedback loop.

These studies are a small part of international endeavours directed at turning an orphan and once dismissed cell into ‘Cinderella’ which may have wide application and unique effects that could cure a number of autoimmune diseases and aid in the prolongation of transplanted tissues.

## Author contributions

All authors have made a substantial, direct and intellectual contribution to the work and approved it for publication.

## Acknowledgments

We would like to thank all authors for their contributions to this Research Topic. We are also like to thank all the reviewers for their time, contributions and improvement of all the manuscripts.

## Conflict of interest

The authors declare that the research was conducted in the absence of any commercial or financial relationships that could be construed as a potential conflict of interest.

## Publisher’s note

All claims expressed in this article are solely those of the authors and do not necessarily represent those of their affiliated organizations, or those of the publisher, the editors and the reviewers. Any product that may be evaluated in this article, or claim that may be made by its manufacturer, is not guaranteed or endorsed by the publisher.
